# Gastropods underwent a major taxonomic turnover during the end-Triassic marine mass extinction event

**DOI:** 10.1371/journal.pone.0276329

**Published:** 2022-11-02

**Authors:** Mariel Ferrari, Michael Hautmann

**Affiliations:** 1 Instituto Patagónico de Geología y Paleontología, IPGP (CCT CONICET-CENPAT), Puerto Madryn, Provincia de Chubut, Argentina; 2 Paläontologisches Institut und Museum, Universität Zürich, Zürich, Switzerland; Naturhistoriska riksmuseet, SWEDEN

## Abstract

Based on an exhaustive database of gastropod genera and subgenera during the Triassic–Jurassic transition, origination and extinction percentages and resulting diversity changes are calculated, with a particular focus on the end-Triassic mass extinction event. We show that gastropods suffered a loss of 56% of genera and subgenera during this event, which was higher than the average of marine life (46.8%). Among molluscs, gastropods were more strongly affected than bivalves (43.4%) but less than ammonoids, which were nearly annihilated. However, there were also pronounced differences among gastropod subclasses. The most strongly affected subclass was the Neritimorphia, which lost 72.7% of their Rhaetian genera; on the other extreme, the Heterobranchia remained nearly unaffected (11% loss). We analysed this extinction pattern with respect to larval development, palaeobiogeography, shell size, and anatomy and found that putative feeding of the pelagic larval stage, adaptation to tropical-temperate water temperatures, and flexibility of the mantle attachment were among the factors that might explain extinction resilience of heterobranchs during the end-Triassic crisis. Among molluscs, extinction magnitude roughly correlates with locomotion activity and thus metabolic rates. We suggest three potential kill mechanisms that could account for these observations: global warming, ocean acidification, and extinction of marine plankton. The end-Triassic extinction of gastropods therefore fits to proposed extinction scenarios for this event, which invoke the magmatic activity of the Central Atlantic Magmatic Province as the ultimate cause of death. With respect to gastropods, the effect of the end-Triassic mass extinction was comparable to that of the end-Permian mass extinction. Notably, Heterobranchia was relatively little affected by both events; the extinction resilience of this subclass during times of global environmental changes was therefore possibly a key aspect of their subsequent evolutionary success.

## Introduction

The end-Triassic mass extinction is commonly ranked among the “big five” extinction events of the Phanerozoic [1] and has attacked considerable scientific attention [2], but surprisingly, there is still a lack of comprehensive studies on the taxonomic turnover for many major taxa. Probably the most prominent example for this lack of knowledge is the gastropods. Gastropods were among the most abundant and taxonomically diverse macrofossils in Triassic–Jurassic (T–J) strata, but their turnover during the end-Triassic crisis has only been analysed in few local studies [3]. Batten [4] suggested that the end-Triassic crisis might have been an even more important caesura in the history of this class than the end-Permian mass extinction, but this claim has never been tested. In this paper, we analyse changes in gastropod diversity at the genus/subgenus level from the Norian to the Pliensbachian, using a taxonomically revised compilation of global occurrences, and assess the relevance and potential causes of the end-Triassic mass extinction for this major class of molluscs.

## Materials and methods

For the construction of the gastropod database, c. 150 original papers that report gastropod occurrences from the analysed interval (Norian–Pliensbachian) were examined (S1 File). Only occurrences that could be verified from figures and detailed taxonomic descriptions were considered. We used the Paleobiology Database (https://paleobiodb.org/#/) and Sepkoski’s [5] database to ensure the completeness of our literature survey.

Original taxonomic assignments were critically assessed and revised when necessary. For the analyses, we applied the range-through assumption, i.e. the assumption that a genus was present in all stages between its first occurrence datum (FAD) and last occurrence datum (LAD) regardless whether it has been recorded continuously or not. However, we retained the information about outages (i.e., absences in the fossil record between the FAD and LAD) for an analysis of the relative completeness of the fossil record.

Based on this data, we constructed an exhaustive presence-absence matrix of gastropod genera from the Norian to the Pliensbachian (S1 Table). From this matrix, we calculated the following parameters for our analyses:

1. *within-bin diversity*: the number of all genera in a given stage, including singeltons and inferred occurrences
2. *boundary crossers*: the number of genera that were present in the stages before and after the stage boundary
3. *originations*: the number of genera that first appeared in a given stage
4. *origination%*: 100*originations/within-bin diversity
5. *extinctions*: the number of genera that have their last appearance datum in a given stage
6. *extinction%*: 100* extinctions/within-bin diversity
7. *simple completeness metric (SCM)*: the ratio of observed occurrences to total occurrences including range-through taxa [6].

For a comparison of the extinction pattern between gastropods and bivalves, we revised and updated the compendium of Ros Franch et al. [7]. This includes the addition of subgenera and occasionally the revision of taxonomic concepts and stratigraphic ranges (S2 Table).

We note that the within-bin diversity approach that we use does not allow to distinguish between sudden changes at the stage boundaries or more gradual transitions over longer time intervals. We therefore refer to the “T–J faunal transition” rather than “T–J mass extinction” in the discussion of our data. However, there is much evidence for a sudden extinction in the latest Triassic from numerous case studies ([2] and refs therein), and we interpret our data in that light.

The literature that underlies the biogeographic analysis is listed in S3 Table.

For assessing the change in shell size, we analysed species of the genus *Cylindrobullina* before and after the extinction event (S4 Table). More precisely, we considered measurements (height of the shell in mm) of 55 specimens belonging to 18 species from the studied time interval, which have originally been assigned to the genus. Data were taken from the literature [8–17] and from available data of the MNHN and GBIF online repositories (https://science.mnhn.fr; https://www.gbif.org).

## Results

**Fig 1:**
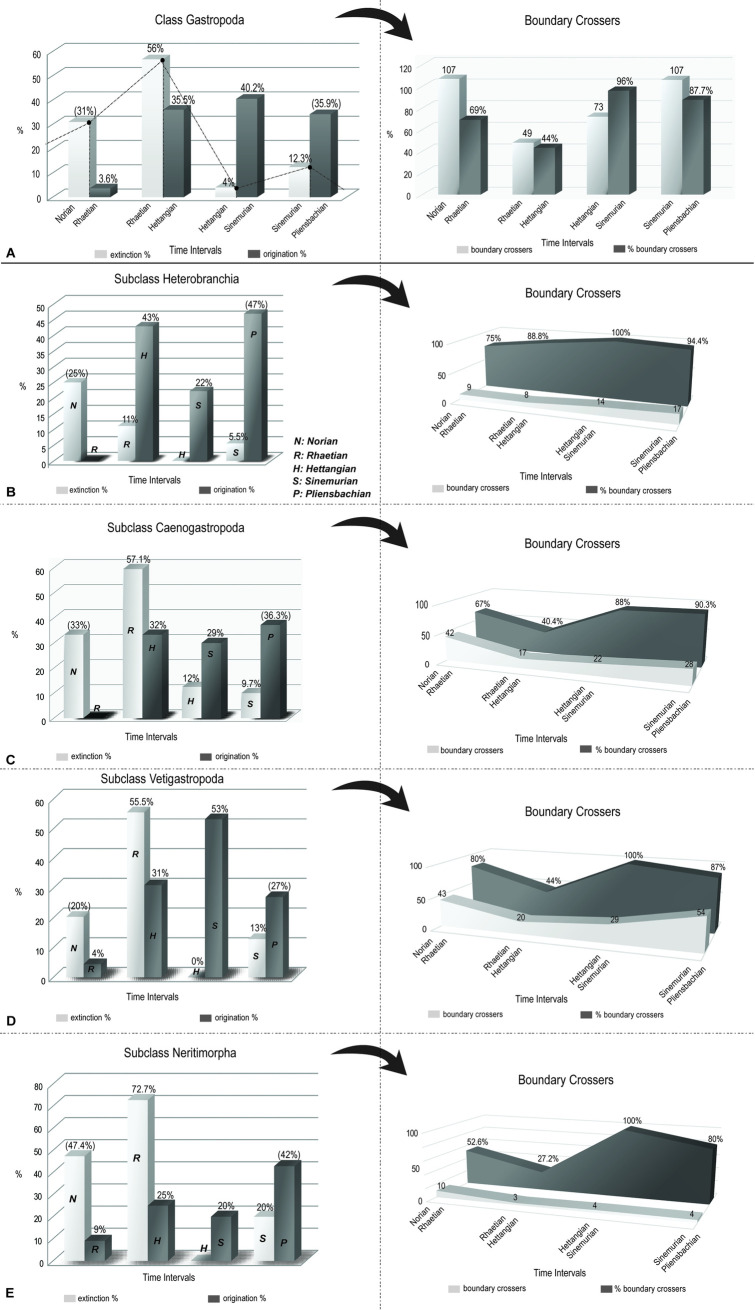
Norian to Pliensbachian extinction and origination percentages and number of boundary crossers for the class Gastropoda and the main subclasses considered in the analysis. **A**, origination and extinction percentages and boundary crossers for the class Gastropoda. The dotted line shows the change of extinction magnitude (%) across the studied time interval. Values in brackets are based on ‘observed within-bin diversity’ and the rest of values on ‘inferred within-bin diversity’. **B**, origination and extinction percentages and boundary crossers for the subclass Heterobranchia. **C**, origination and extinction percentages and boundary crossers for the subclass Caenogastropoda. **D**, origination and extinction percentages and boundary crossers for the subclass Vetigastropoda. **E**, origination and extinction percentages and boundary crossers for the subclass Neritimorpha.

The number of boundary crossers among gastropod genera dropped dramatically during the end-Triassic mass extinction, from 107 genera at the Norian–Rhaetian boundary to merely 48 genera at the T–J boundary (Fig 1A). This translates into a 56% extinction at the genus level (Fig 1A), which is much higher than the 43.4% extinction of bivalve genera (see below) and the average of 46.8% for all marine genera [18]. Yet, high post-extinction origination (27 newly evolved genera in the Hettangian, corresponding to 35.5% origination) led to a relatively quick compensation of a significant part of this loss. Hettangian within-bin diversity (76 genera) was therefore not so different from Rhaetian within-bin diversity (111 genera), and Sinemurian within-bin diversity (122 genera) already exceeded the pre-extinction level. This trend continued into the Pliensbachian (162 genera; Table 1).

**Table 1:** Summary of data.

	Norian	Rhaetian	Hettangian	Sinemurian	Pliensb.
**Gastropoda, all genera**					
(sub)genera (inferred)	--	111	76	122	--
(sub)genera (observed)	155	107	57	115	161
originations	--	4	27	49	55
extinctions	48	63	3	15	--
SCM	--	96.4%	75%	94.2%	--
**Heterobranchia**					
(sub)genera (inferred)	--	9	14	18	--
(sub)genera (observed)	12	9	11	17	32
originations	--	0	6	4	15
extinctions	3	1	0	1	--
SCM	--	100%	78.6%	94.4%	--
**Caenogastropoda**					
(sub)genera (inferred)	--	42	25	31	--
(sub)genera (observed)	63	41	16	27	43
originations	--	0	8	9	16
extinctions	21	25	3	3	--
SCM	--	97.62%	64%	87%	--
**Vetigastropoda**					
(sub)genera (inferred)	--	45	29	62	--
(sub)genera (observed)	54	44	21	59	74
originations	--	2	9	33	20
extinctions	11	25	0	8	--
SCM	--	97.78%	72.41%	95%	--
**Neritimorpha**					
(sub)genera (inferred)	--	11	4	5	--
(sub)genera (observed)	19	10	4	5	7
originations	--	1	1	1	3
extinctions	9	8	0	1	--
SCM	--	90.9%	100%	100%	--

Inferred (based on the range-through assumption) and observed numbers of genera and subgenera (= within-bin diversity) per stage. Based on these values, the number of originations and extinctions, and Simple Completeness Metrics (SCM) are calculated for the class Gastropoda and its four most diverse subclasses.

Dissecting the diversity trend into its origination and extinction components shows that the decrease of within-bin diversity from the Norian (155 genera) to the Rhaetian (111 genera) was essentially driven by a lack of origination in the Rhaetian (Fig 1A; Table 1). In contrast, the diversity decline at the end of the Triassic was driven by dramatically increased extinction (Fig 1A; Table 1). Thus, the suggestion of Bambach et al. [18] that low origination contributed more than high extinction to the marked loss of diversity in this event is true for the Norian–Rhaetian decline in gastropod diversity, but not for the end-Triassic gastropod extinction event itself.

Comparing the diversity trajectories among the different subclasses of gastropods revealed both commonalities and differences. In the studied interval, marine gastropods were represented by six subclasses; these are Caenogastropoda, Vetigastropoda, Heterobranchia, Neritimorpha, Patellogastropoda, Amphigastropoda, and genera grouped in a basal ancestral position.

The Subclass Heterobranchia comprises the clades “Lower Heterobranchia”, Cohort Acteonimorpha and taxa of uncertain position [19]. This clade suffered merely ~11% extinction at the end of the Triassic, whereas the origination percentage of this clade during the Hettangian was high (~43%) and increased further in the Pliensbachian (~47%; Fig 1B).

The Subclass Caenogastropoda contains the Superorder Latrogastropoda, Order Neogastropoda, Cohort Sorbeoconcha, subcohorts Hypsogastropoda, Campanilimorpha, Cerithiimorpha, and taxa of uncertain position [19]. Caenogastropoda was strongly affected by the Late Triassic crisis with a loss of ~57% of Rhaetian genera (Fig 1C). Caenogastropoda was also the only subclass that suffered additional extinction in the Hettangian (~12%). On the other hand, the origination within this clade was ~32% in the Hettangian and ~36.3% in the Pliensbachian, indicating recovery from the extinction event, although pre-extinction diversity was not reached in the studied time interval.

The Subclass Vetigastropoda includes the orders Lepetellida, Pleurotomariida, Seguenziida, Trochida and taxa of uncertain position [19]. Vetigastropoda suffered elevated extinction during the Rhaetian, with a loss of ~55.5% of genera (Fig 1D). The origination percentage of vetigastropods was ~31% during the Hettangian and increased up to 53% during the Sinemurian. In the Pliensbachian, the origination percentage of vetigastropods dropped down to ~27%.

Finally, the subclass Neritimorpha suffered the highest extinction percentage during the T–J transition, with a ~ 72% extinction of Rhaetian genera (Fig 1E). Extinction was followed by a very slow recovery during the Hettangian with ~25% origination.

Patellogastropoda, Amphigastropoda and clades of Basal Ancestral Position were excluded from detailed analyses of the extinction and origination pattern because of the low number of genera within the studied time interval.

The diversity trajectories reveal the lack of any origination in the Rhaetian for the subclass Heterobranchia and Caenogastropoda, whereas Vetigastropoda and Neritimorpha show minor origination at that time. On the other hand, the diversity trajectories agree in the lack of any extinction (except for Caeonogastropoda with 12%) during the Hettangian. Moreover, origination continued to exceed extinction for all subclasses until the end of the studied time interval.

However, there are also differences among subclasses. As already mentioned, some genera of Caenogastropoda became extinct during the Hettangian, whereas the other subclasses did not suffer any extinction in this stage. Among the three genera of Caenogastropoda that went extinct in the Hettangian, *Palaeorissoina* and *Bourguetia* were Hettangian singletons [8, 20, 21], whereas *Angularia* is an example of “dead clade walking” [22]. This means that *Angularia* survived the end-Triassic mass extinction but disappeared during the Hettangian [8, 9–23]. Other examples of dead clade walking are *Bandelopsis* (subclass Neritimorpha) and *Amphitrochu*s (subclass Vetigastropoda), which re-appeared in the Sinemurian.

The subclass Heterobranchia differs from all other subclasses in that the extinction intensity during the end-Triassic extinction (11.1%; Fig 1B) was lower than at the end of the Norian (25%) and dramatically lower than the average of the class (56%). Moreover, Heterobranchia also had among the highest originations in the Hettangian and the following stages. In combination, Heterobranchia increased its diversity absolutely and relative to other subclasses during the T–J faunal turnover.

**Fig 2:**
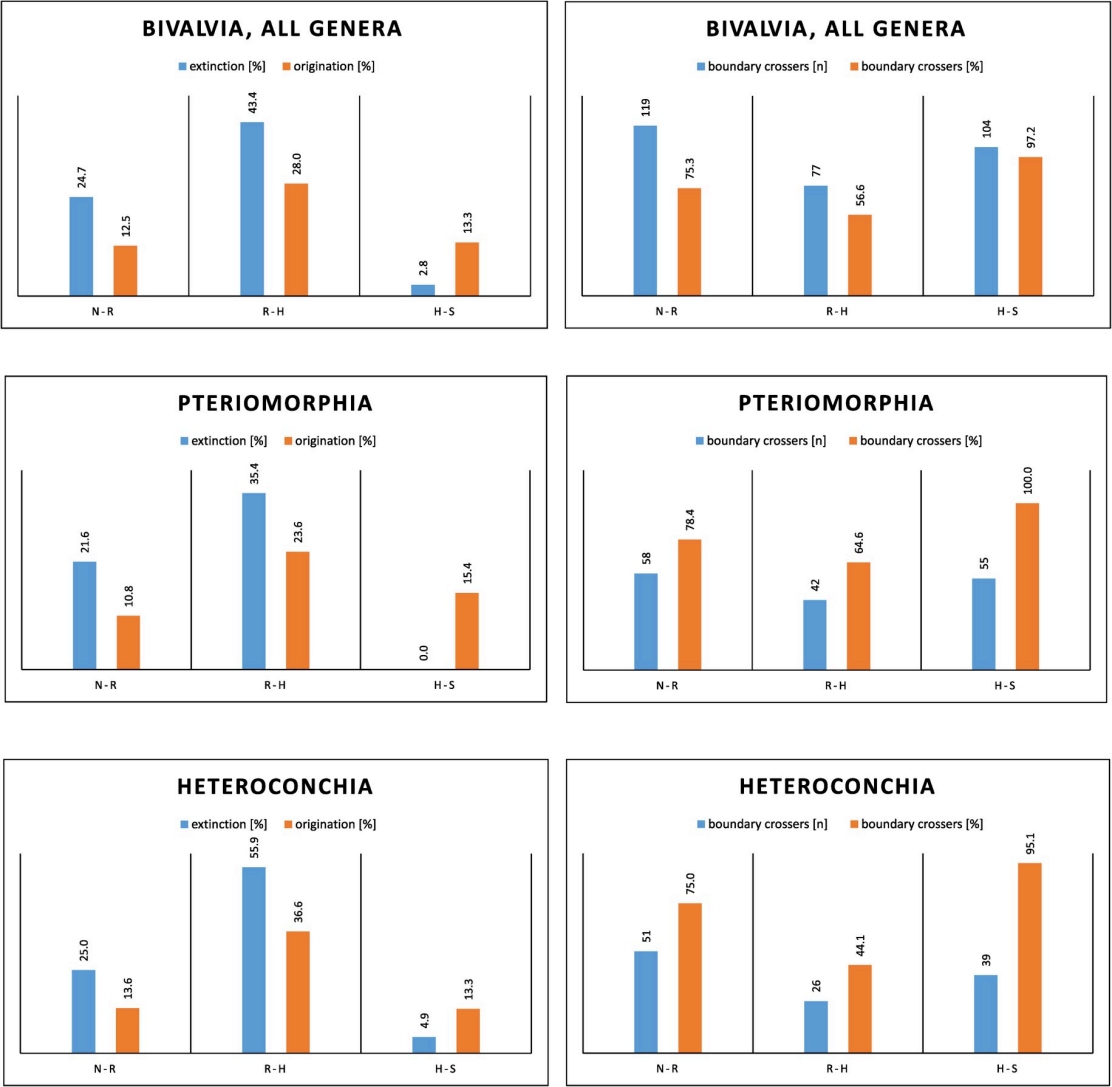
Extinction and origination percentages and boundary crossers of bivalve genera from the Norian–Sinemurian. **A, B,** all genera; **C, D,** Pteriomorphia; **E, F,** Heteroconchia.

Bivalves (S2 Table) suffered relatively lower extinction (43.4%) than gastropods during the T–J transition, followed by increased origination (28%) and decreased extinction (2.8%) in the Hettangian (Fig 2A). As in gastropods, there were also differences in the pattern between the major subclasses (Fig 2C–2F). Heteroconchia suffered higher extinction (55.9%) than Pteriomorphia (35.4%; χ^2^ = 5.27, p = 0.02) but also higher post-extinction origination in the Hettangian (36.6% versus 23.9%; χ^2^ = 1.91, p = 0.18). In the post-extinction diversity dynamics, Pteriomorphia were less volatile than Heteroconchia, which is indicated by the absence of genus-level extinction in the Hettangian and the higher percentage of early Jurassic boundary crossers.

## Discussion

### Effects from differential completeness

**Fig 3:**
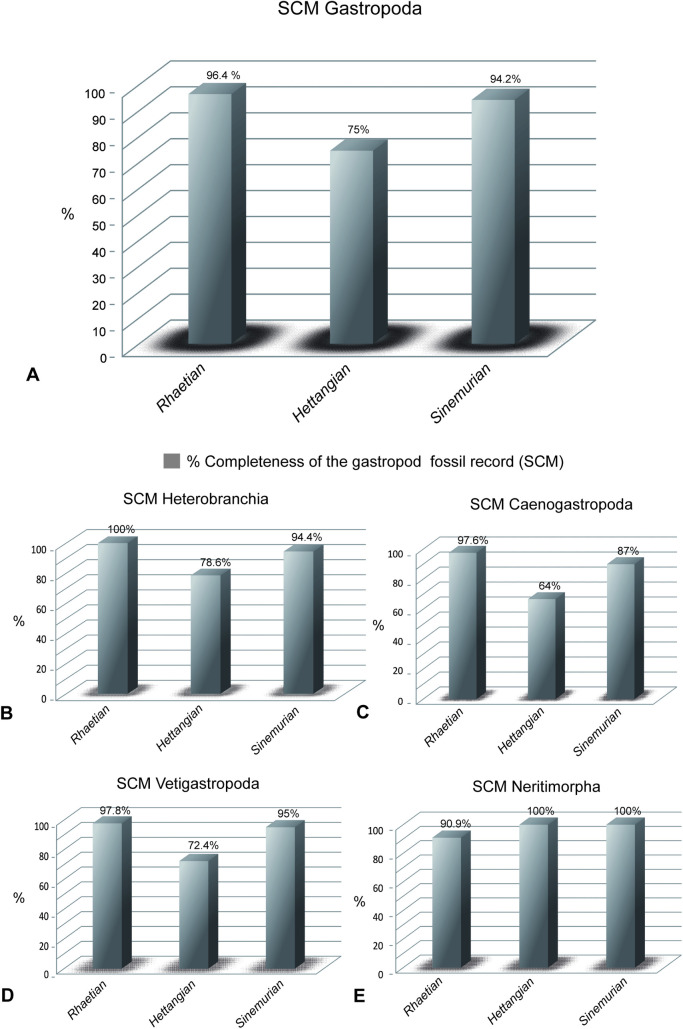
Percentage of completeness (SCM) of the gastropod fossil record during the T-J boundary. **A,** SCM of the Class Gastropoda. **B**, SCM of Heterobranchia. **C**, SCM of Caenogastropoda. **D**, SCM of Vetigastropoda. **E**, SCM of Neritimorpha. See text for details.

The SCM data (ratio of observed occurrences to total occurrences including range-through taxa; Fig 3; Table 1) indicate that the fossil record was less complete in the first stage after the extinction event than in the preceding and following stages. The relatively low Hettangian SMC value corresponds to a moderate decline in the number of collections reported by Kiessling et al. ([24], Fig 1, Table 1) for this stage, based on data from the PBDB. This underrepresentation may simply result from the shorter duration of the Hettangian (~2 Ma [25] to maximum ~4.7 Ma [26]) in comparison to the Rhaetian (7.2 Ma) and Sinemurian (8.5 Ma; [25]).

Although the lower Hettangian completeness potentially overemphasizes the extinction magnitude, the observed decline in SCM by 21.4% (from 96.4% to 75%) is much lower than the observed extinction magnitude of gastropods (56.%), which was therefore not an artefact of differential completeness.

SCM differs among subclasses (Fig 3B–3E); in the Hettangian, it ranges from 100% in the Neritimorpha to 64% in the Caenogastropoda. Notably, this pattern does not translate into systematic differences in the observed T–J extinction magnitudes: We found the highest extinction magnitude for the Neritimorpha (72.7%), although this subclass had the highest post-extinction SCM (100%). The differences in SCM among the other subclasses are only moderate (78.6%– 64%) and insufficient to explain the much larger differences in the extinction magnitudes (Table 1).

### Similarities and differences in extinction-recovery patterns among subclasses

Analysing the diversity dynamics of gastropods across the T–J transition shows some aspects that are not unexpected. This includes the increase in origination and decrease in extinction in the stage after the main extinction, a phenomenon that is expected and has also been observed in the wake of other mass extinctions [27]. However, there are also differences among gastropod subclasses that require further explanation. Most notably, Heterobranchia suffered less extinction and higher post-extinction origination than all other subclasses (~11% genus-level extinction at the end of the Triassic and ~43% of origination in the Hettangian; see above). Chi square tests (Table 2) confirm that the difference to the other subclasses are statistically significant.

**Table 2:** Chi square test statistics for differences in extinction magnitude among gastropod subclasses.

	Heterobranchia	Caenogastropoda	Vetigastropoda	Neritimorpha
**Heterobranchia**	--	**χ**^**2**^ **= 9.7767**	**χ**^**2**^ **= 4.86**	**χ**^**2**^ **= 5.6902**
		**p = 0.002**	**p = 0.027**	**p = 0.017**
**Caenogastropoda**	**χ**^**2**^ **= 9.7767**	--	0.049809	χ^2^ = 0.36533
	**p = 0.002**		p = 0.823	p = 0.546
**Vetigastropoda**	**χ**^**2**^ **= 4.86**	χ^2^ = 0.049809	--	χ^2^ = 0.55753
	**p = 0.027**	p = 0.823		p = 0.455
**Neritimorpha**	**χ**^**2**^ **= 5.6902**	χ^2^ = 0.36533	χ^2^ = 0.55753	--
	**p = 0.017**	p = 0.546	p = 0.455	

Significantly different pairs (p < 0.05) are indicated in bold.

Because it is difficult to infer the ecology of extinct gastropods from the shell morphology, indirect lines of evidence are required to understand the relative resilience of Heterobranchia during the end-Triassic crisis. Here, we discuss (1) larval development, (2) palaeogeographic distribution, (3) shell size, and (4) putative anatomical features inferred from recent representatives and phylogeny.

#### Larval development

Larvae of gastropods might be planktotrophic (feeding on plankton) or non-planktotrophic (feeding from egg [lecithotrophic] or having a direct development [28]) In fossil marine gastropods, planktotrophy or nonplanktotrophy can be inferred from the dimensions and the morphology of the protoconch (larval shell). The protoconch of gastropods consists of a protoconch I (embryonic shell) that is formed within the egg prior to hatching, and a protoconch II that is formed after hatching. Gastropods with planktrophic larvae have typically a smaller embryonic shell (size of first whorl) and a higher number of protoconch whorls than non-planktotrophic gastropods [28]. The herein discussed subclasses except vetigastropods had planktotrophic larvae [28], but the duration of the larval stage might have differed. Nützel [28] pointed out that Heterobranchia typically have larval shells with smaller and fewer whorls (less than 2.5) than Caenogastropoda (up to 10 whorls), and that the number of protoconch whorls is negatively correlated with the diameter of the first whorl (embryonic shell) and positively correlated with the duration of the pelagic period. Thus, Heterobranchia probably have had a relatively shorter pelagic period than Caenogastropoda. Haszprunar [29] suggested that the types of heterostrophy, which may be transaxial (twisted 90° or perpendicular to the shell axis of the teleoconch) or coaxial (twist 180° or relatively parallel to the shell axis of the teleoconch), appear to be correlated with the duration of the pelagic period. Accordingly, if the pelagic phase is relatively long, the protoconch is twisted 90° (transaxial), and if the pelagic period is relatively short, it is twisted 180° (coaxial) [29]. In the case of the Late Triassic Heterobranchia, both representatives of the “Lower Heterobranchia” (e.g. *Promathildia*) and the Acteonimorpha group (*Consobrinella*, *Sinuarbullina*, *Domerionina*) had a transaxial hererostrophic protoconch, whereas other basal cylindrobullinids (e.g. *Cylindrobullina*) had a coaxial to transaxial protoconch. This suggests that the style of heterostrophy and accordingly the duration of the pelagic larval phase was variable within the basal heterobranchids. In spite of this variation, even species of the transaxial group likely had a shorter pelagic larval phase than caenogastropods because of the much lower number of protoconch whorls (see above).

The duration of the planktotrophic larval stage affects a number of potentially extinction-relevant factors such as gene flow, dispersal ability and size of geographic distribution (e.g., [30]). Usually, a wider geographic distribution buffers against extinction risk, but such an effect was not observed for the end-Triassic mass extinction event [31]. Our data confirm this unusual pattern, because the shorter larval stage in Heterobranchia suggests a more restricted geographic distribution (as confirmed by our data; see below) and thus a higher extinction risk in comparison to e.g. Caenogastropoda, and not the *lower* extinction magnitude that is actually observed.

An alternative explanation could be that gastropod larvae with a longer pelagic phase had different nutrition requirements than larvae that remain smaller. Small gastropod larvae feed on phytoplankton and nannoplankton, and often uptake dissolved organic matter and detritus, whereas larvae with a long pelagic stage that grow larger (as in Caenogastropoda) are feeders on zooplankton (Radiolaria, Foraminifera), fish eggs, copepods, diatoms and dinoflagellates [28]. Unfortunately, data on plankton turnover during the end-Triassic crisis are limited. Ward et al. [32] suggested a sudden productivity collapse and plankton extinction, based on T–J sections in British Columbia; Clémence et al. [33] reported a short-term bloom of schizosphaerellids in the wake of the extinction in sections of SW England; Schootbrugge et al. [34] documented a change from chromophyte- to chlorophyte-dominated phytoplankton assemblages across the T–J on the European continental shelf. Although we know little about global diversity changes of plankton or the fate of zooplankton across the T–J transition, it seems possible that the ability to feed on non-living matter such as dissolved organic matter and detritus was advantageous during a mass extinction event that potentially affected planktonic organisms.

#### Geographic distribution

**Fig 4:**
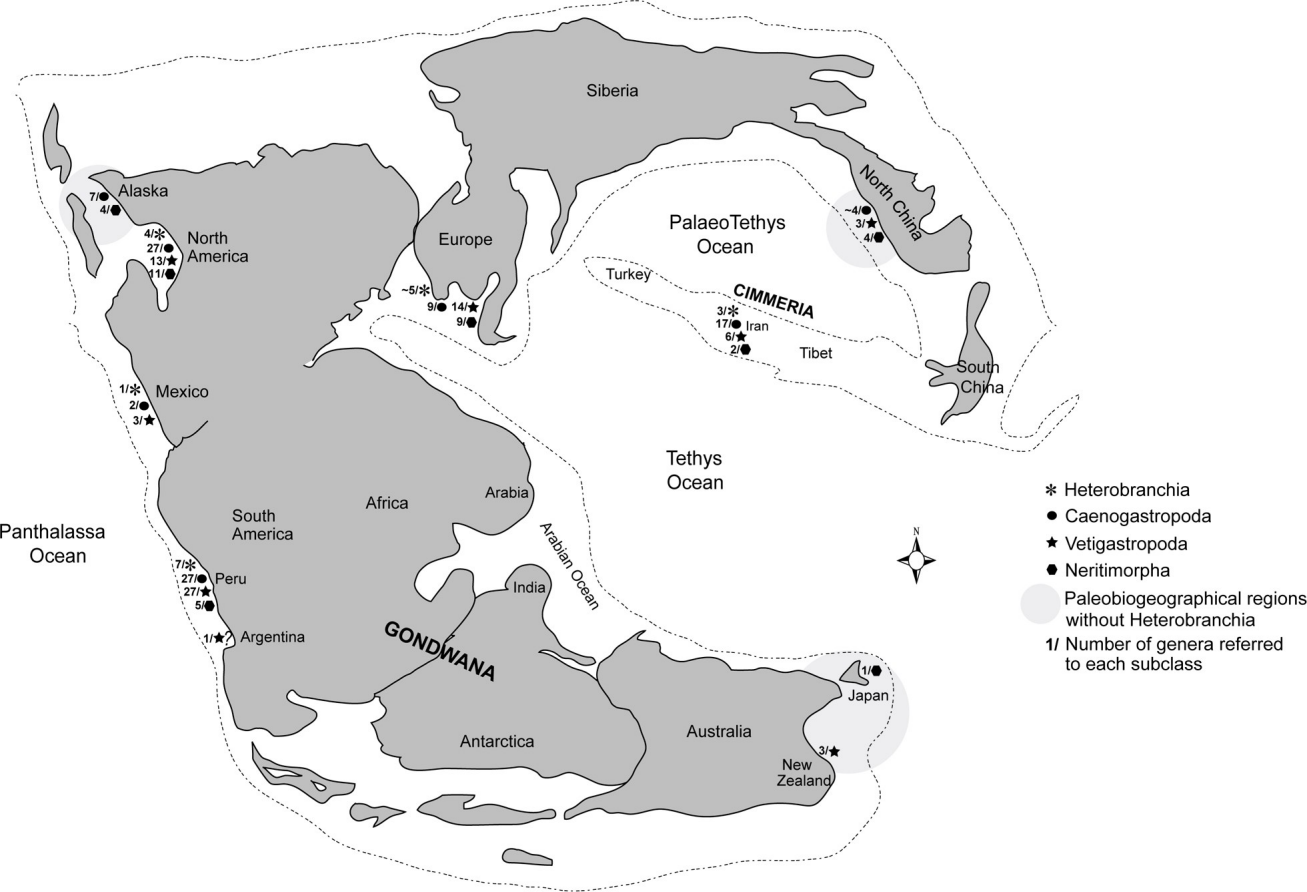
Palaeobiogeographical map showing the pre-extinction distribution of the main gastropod subclasses during the Late Triassic (Norian-Rhaetian; 227–201.3 Ma). Subclasses, the number of genera referred to each subclass, and the palaeobiogeographical regions without members of Heterobranchia are indicated (Data: S3 Table).

The main gastropod subclasses discussed herein had a relatively wide palaeogeographic distribution during the Norian-Rhaetian and were typical components of the benthic marine communities before the T–J extinction (Fig 4). Gastropod faunas have been reported along the entire eastern margin of the Paleo-Pacific Ocean, from Alaska [35–38], via North America and Mexico [23, 39–42] to the South American Andes in Peru [10] and probably the Patagonian region in Argentina [43]. They also occurred along the Paleo-Tethyan margins of Iran [9, 44–46], north western China [47, 48], western Tethyan region [49–53], New Zealand [54] and Japan [55] (Fig 4).

However, details of the palaeogeographic distribution of the subclasses reveal that gastropods were subject to a latitudinal effect, with a lower number of subclasses in higher palaeolatitudes, including the invariant absence of the Heterobranchia in the boreal realm (Alaska, Japan–New Zealand, and North China; Fig 4). The more restricted biogeographic distribution of the Heterobranchia in comparison to other subclasses might be related to a shorter larval stage (see above), but their absence from higher palaeolatitudes suggests that palaeotemperature was an additional factor that controlled the distribution of this subclass. The palaeobiogeographic pattern is at variance with a cooling event (e.g., a volcanic winter scenario), because cooling should have affected tropical to temperate taxa more severely than taxa that extended into the boreal. However, the pattern is consistent with global warming if we assume that the Late Triassic absence of Heterobranchia from high palaeolatitudes reflects an adaptation to warm temperatures, which could have made heterobranchs less vulnerable to global warming and/or allowed them to survive by a migration towards higher palaeolatitudes when global temperatures increased. This interpretation is consistent with a spread of heterobranchs to higher latitudes in the Early and Middle Jurassic, when some genera (e.g. *Tricarilda*, *Cristalloella*, *Camponaxis*, *Consuella*, *Sulcoactaeo*n, *Promathildia*; see [56] and [11]) reached high latitudes in the austral region of New Zealand.

The case of the Heterobranchia also sheds light on the differential effect on the extinction risk that geographic distribution has during background times versus mass extinction events. Jablonski [57] found that wider geographic ranges correlate with lower extinction risks in background times but not during the end-Cretaceous mass extinction. Kiessling and Aberhan [31] came to a similar conclusion for the end-Triassic mass extinction event, which according to their data affected geographically restricted and widespread genera equally. Generally, geographic distribution seems to be strongly correlated with extinction risk in background times but not during mass extinctions [58]. The example of Heterobranchia during the end-Triassic mass extinction event is conform to the explanation that Payne and Finnegan [58] provided for this phenomenon. These authors suggested that widespread environmental disturbances during mass extinctions simultaneously affected genera with similar ecological and physiological characteristics on global scales. Clades with a restricted geographic distribution might therefore survive if their physiology buffers against the kill mechanism, whereas widespread clades without this tolerance might go extinct if the change was global in extent and therefore did not leave any refugia.

### Size

**Fig 5:**
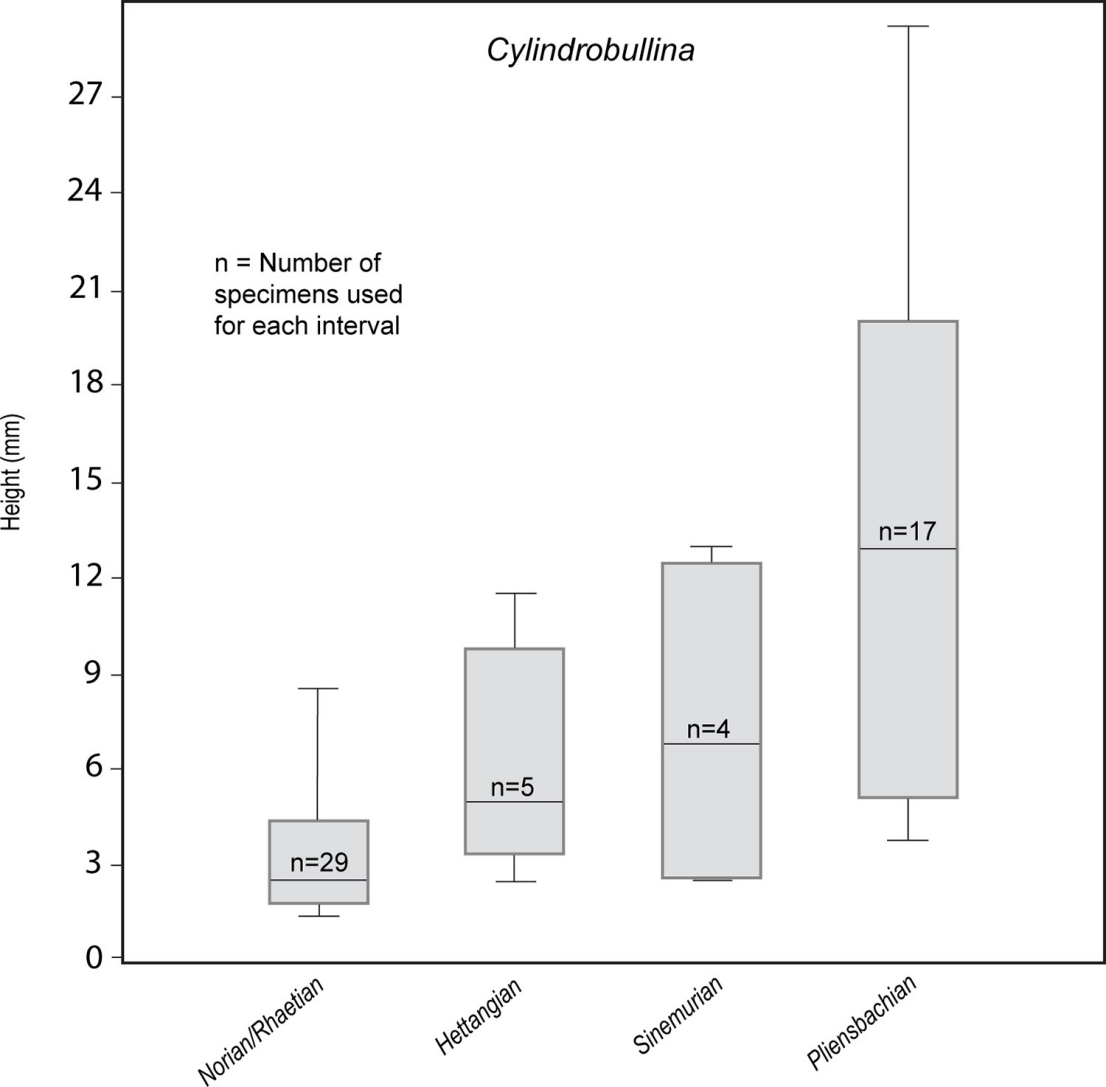
Shell size (box plot) of *Cylindrobullina* during the Triassic–Jurassic transition. Note the increase in shell size (height in mm) from the Norian–Rhaetian to the Pliensbachian. Horizontal lines: mean; boxes: 25–75 percent quartiles; vertical lines: minimum and maximum values.

Mass extinctions are often selective for body size (e.g., [59]). However, body size depends on ontogenetic stages (juveniles are smaller than adults) and phylogenetic constrains (some taxa grow to larger sizes than others). We therefore restrict our size analysis to species of a single genus (*Cylindrobullina*) before (Norian–Rhaetian) and after (Hettangian–Pliensbachian) the extinction, using only specimens for which an adult age could be verified. *Cylindrobullina* has been chosen because it is the only gastropod genus for which sufficient data of pre- and post-extinction shell sizes are available. *Cylindrobullina* is the type genus of the family Cylindrobulinidae and was abundant and diverse during the Late Triassic and Early Jurassic. The analysis revealed an increase in maximum shell size throughout the entire interval including the extinction event (see S4 Table): Norian–Rhaetian, maximum height < 9 mm; Hettangian, maximum height up to 11.5 mm; Sinemurian, maximum height up to 13 mm; Pliensbachian, maximum height up to 30 mm (Fig 5).

The observed increase in maximum shell size is a phenomenon that is opposite to the frequently cited Lilliput effect [60, 61 and refs therein], which refers to a decrease in size after a mass extinction event. However, our observation is not isolated. Brayard et al. [62, 63] reported ‘Gulliver gastropods’ that question the Lilliput effect in the wake of the end-Permian mass extinction. More recently, Atkinson et al. [64] have described a post-extinction size increase from Early Jurassic bivalves of England and referred to it as the ‘Brobdingnag Effect’. They suggested that this size increase might have been caused by changes in water depth, oxygen content, food availabilities, or temperature. However, all these features vary locally and are thus unlikely to have had an interregionally uniform effect, as observed in our data (S4 Table). The Late Triassic (Norian-Rhaetian) *Cylindrobullina* species in our dataset are mostly from Peru (Andes of South America) and they show the smallest size for the genus at that time. Cylindrobullinids with a medium size were mostly found in the western Tethyan region during the Hettangian to Pliensbachian, and largest representatives of *Cylindrobullina* occurred in the Pliensbachian of southern South America (Argentina) (S4 Table). The only possible exception is the Pliensbachian size increase, which might be related to a late Pliensbachian cooling event, as proposed by Schootbrugge et al. [65] and Korte et al. [66]. Moreover, the largest specimens of *Cylindrobullina* (height up to 30 mm) in our data stem from the Pliensbachian of high palaeolatitudes (south Patagonia, southernmost South America), with inferred cold palaeotemperatures. However, there is no correlation between palaeolatitude and the shell size of *Cylindrobullina* in the other stratigraphic stages. As an alternative, the post-extinction size increase could have resulted from ecological opportunities due to the increased extinction of large taxa that previously competed with *Cylindrobullina*. Testing this hypothesis requires a dataset of sizes of potentially competing taxa before and after the extinction, which is currently not available for the reasons outlined above and therefore remains a future task.

#### Anatomy

The anatomy of Late Triassic–Early Jurassic Heterobranchia has to be inferred indirectly from Recent members of the subclass and phylogenetic considerations and is therefore partly speculative. Early Mesozoic Heterobranchia is represented by the “Lower Heterobranchanchia” and Acteonimorpha clade (see above). According to the recent literature [19, 67–70], these basal heterobranchids were possible ancestors of the extant Euthyneura. The phylogenetic study of Kano et al. [70], which combined fossil evidence with anatomical and molecular data, showed that the stem group of Euthyneura (or “shelled opisthobranchs”) first existed in the earliest Triassic (~250 Ma). This conclusion is supported by the phylogenetic analysis of Dinapoli [69], who indicated an occurrence of the Euthyneura stem line between the Mid Carboniferous and the Early Triassic and a first diversification of the clade between the Permian and the Triassic. The early Mesozoic euthyneuran bubble snails of the extinct families Cylindrobullinidae, Tubiferidae and Acteonellidae are very similar in general shell morphology and ornamentation pattern to the extant Acteonidae and Ringiculoides (see Kano et al. [70], Fig 3). Thus, the common ancestor of Euthyneura might have had a thin, oval shell-shaped with a large body whorl, an elongated aperture and smooth surface with or without fine spiral cords, as is shown in early Mesozoic opisthobranchids (e.g., *Cylindrobullina*, *Conactaeon*, *Euconactaeon*, *Sinuarbullina*). Ecologically, these characters typically occur in species with an infaunal or temporarily borrowing mode of life [70]. A large and elongated aperture is commonly associated with the presence of a large foot that enables rapid and efficient burrowing, as does the hypertrophied head (headshield), which is typical of the extant ringiculids (see Kano et al. [70], Fig 3). Kano et al. [70] also argued that infaunal snails bear an expanded mantle that partly or entirely covers the shell for further facilitating locomotion. If this character was present among the fossil heterobranchs discussed herein, it could have contributed to the resilience against the extinction event for two reasons. First, an expanded mantle that covered much part of the shell would be advantageous during ocean acidification (see below), because it protects the shell against dissolution and facilitates shell secretion in an environment with reduced CaCO_3_ saturation. Second, the relaxed connection between the mantle margin and shell lip released the mantle from morphological constraints and might have led to an increased flexibility of the body plan that facilitated evolutionary innovations [70]. Kano et al. [70] regarded the evolutionary flexibility of Heterobranchia chiefly as “a key preadaptive trait behind the explosive Mesozoic radiation of Euthyneura”, but it could likewise have allowed heterobranchs to respond evolutionarily more quickly than other gastropod subclasses to sudden environmental changes during mass extinctions.

### Comparison with other molluscs

Within the Mollusca that have a well-documented fossil record, the extinction of gastropod genera/subgenera (56.8%) is in a medial position. Gastropods were less affected than ammonoids, which suffered nearly complete annihilation [71], but clearly more strongly affected than bivalves with merely 43.4% extinction at the genus/subgenus level (this study; Fig 2, S2 Table). This seems to be related neither to the benthic versus nektonic mode of life nor to the position in the food web, because in these cases bivalves and gastropods should have had similar extinction risks. However, the increasing extinction magnitude from bivalves via gastropods to ammonoids corresponds to increasing mobility and thus, by inference, increasing metabolic rates. The majority of bivalves is stationary or only slowly moving, although exceptions of very active taxa exist that are discussed below. In contrast, gastropods are usually crawling, and ammonoids were more or less active swimmers. These different locomotion activities required increasingly more energy. A possible explanation for the observed correlation between extinction magnitude and locomotion activity could be that CO_2_ emissions from the Central Atlantic Magmatic Province (CAMP) [72, 73] led to a temperature rise (but see [74]) and a corresponding decrease of dissolved oxygen in the seawater, which put active organisms with high metabolic rates at an elevated extinction risk.

As noted above, the majority of bivalves are stationary or slowly/sporadically moving organisms, but there are also very active taxa, which allow for testing the hypothesis that species with more active lifestyles were at an elevated extinction risk during the end-Triassic crisis. Among the bivalves with the highest locomotion activity in the Mesozoic were the trigoniids [75, 76]. Mesozoic trigoniids typically occur in nearshore habitats with coarse, shifting substrata that require adaptations for high burrowing rates [67]. We reviewed Rhaetian genera of the order Trigoniida, which includes the superfamilies Trigonioidea sl. and Myophorioidea, with respect to shell morphological adaptations for high burrowing rates (see Stanley [75]). On this basis, we excluded two genera (*Costatoria* and *Neoschizodus*) from the list of fast-burrowing genera in the Trigonoida. Among the remaining nine genera and subgenera, all but one (*Prosogyrotrigonia*; S2 Table) went extinct at the end of the Triassic. This corresponds to c. 89% extinction in this group, which is much higher than the average for bivalves (43.4%). Although the number of trigonoid genera is relatively small, the difference is statistically significant (χ^2^ = 6.3, p = 0.01), which supports the hypothesis that the end-Triassic mass extinction selected against organisms with high motility and a correspondingly high energy consumption.

A commonality of gastropods, bivalves and ammonoids is their relatively quick recovery. As noted above, 27 gastropod genera newly evolved during the Hettangian, corresponding to 36%% origination. In bivalves, there are 30 genera that first appeared in the Hettangian, corresponding to still impressive 28% origination (S2 Table). A quick recovery of bivalves after the end-Triassic mass extinction has also been observed in local palaeo-communities in Tibet [77] and Britain [78]. The quick Hettangian recovery of ammonoids is also well-established (e.g., [71, 79]).

The quick recovery of the benthic mollusc classes Gastropoda and Bivalvia is in contrast to their decidedly delayed recovery from the end-Permian mass extinction. Apart from possible environmental causes; differences in the extinction magnitude and the intensity of interspecific competition may explain this pattern [80].

### Causes of extinction

There is an increasing consensus that the end-Triassic mass extinction was caused by a number of environmental changes that resulted from the contemporaneous volcanism of the Central Atlantic Magmatic Province (CAMP); these include climatic changes, ocean acidification, sea level changes, marine anoxia, and the emission of toxic elements and compounds (see summary in [2]). Our data allow for testing the first two of these possible causes.

#### Ocean acidification

Ocean acidification may harm marine organisms for a variety of reasons (e.g., [81, 82]). Some potentially detrimental effects refer to aspects of the soft body that are difficult to reconstruct in palaeontological data, but ocean acidification has also a profound negative effect on calcareous biomineralization (e.g., [83]), which can be tested with data from shelly fossils. Accordingly, taxa with aragonitic shells should suffer more than taxa with calcitic shells during an ocean acidification event [84, 85]. Ocean acidification as a kill mechanism is therefore in accordance with the above-average extinction percentage of gastropods, which had invariably aragonitic shells.

The low extinction percentage of subclass Heterobranchia in comparison to other gastropod subclasses is more difficult to explain in the context of an ocean acidification scenario. There is no evidence that heterobranchs differed from other gastropods in their shell mineralogy, but the heterobranchids (including also the basal euthyneuran) seem to have had a unique anatomical feature in the Triassic that might have been advantageous during times of low seawater pH. They developed an expanded mantle that partly or entirely covered the shell [70], and coverage of the shell by this expanded mantle could have protected it from dissolution in an environment that is undersaturated with respect to CaCO_3_, and it would also be advantageous when new shell is formed in an undersaturated environment (see above). In addition, the potentially infaunal mode of life of heterobranchs [70] provided a chemical setting that differed from the seawater and could have buffered against acidification, particularly where the sediment was calcareous.

#### Temperature changes

Theory predicts that massive volcanism may cause a short-term cooling through the emission of SO_2_ that rapidly forms sulfate aerosols in the atmosphere that absorb or backscatter sunlight, followed by a longer period of global warming through CO_2_ emissions [86]. Although empirical evidence for temperature changes is controversial [75, 87], a rise in palaeo-CO_2_ concentrations is well established, suggesting maximum concentrations between 2750 ppmv [88] and 4400 ppmv [89]. As discussed above, the preferred survival of heterobranchs correlates with a tropical to temperate pre-extinction palaeobiogeography and thus a warm-water affinity. Heterobranchs were therefore likely preadapted to increasing temperatures and could also avoid extinction through migration towards higher latitudes after the end-Triassic crisis.

Because dissolved oxygen decreases with seawater temperature, a warming event might also underlay the increasing extinction magnitude of mollusc classes that are more active and therefore require more oxygen. Accordingly, higher motility and thus higher energy demands translate into the increased extinction magnitude that is observed from bivalves via gastropods to ammonoids. As discussed above, this effect is confirmed within bivalves, where particularly active bivalve taxa suffered much higher extinction than the average of this class.

### Comparison with the end-Permian mass extinction event

Batten [4] suggested that the end-Triassic mass extinction event might have been an even more important caesura in the history of gastropods than the greatest Phanerozoic mass extinction event at the end of the Permian. Here we reassess this claim by comparing our results for the end-Triassic event with published data for the Permian–Triassic (P–T) transition. It should be noted that no comparable quantitative analysis for the end-Permian mass extinction exists, but Nützel [90] provides a very informative overview of the main patterns across the Permian–Triassic transition, which allows at least a qualitative comparison. Accordingly, some gastropod subclasses responded similarly to the end-Permian and end-Triassic mass extinction events, whereas others showed different patterns.

Examples of similar patterns include the vetigastropods and caenogastropods, which were strongly affected by both events, and caenogastropods, which had a relatively rapid recovery in both post extinction intervals. The fossil record of the Heterobranchia in the Early Triassic is relatively poor, but several genera crossed the P–T boundary and contributed considerably to the recovery process through the origination and surprisingly early radiation of the opisthobranchs (cylindrobullinids) and the later origination of the mathildids [90]. Thus, the Heterobranchia seem to have eventually benefitted from both extinction events, which could have catalyzed their evolutionary success.

In contrast, Vetigastropoda had a quicker recovery during the earliest Jurassic than in the earliest Triassic, and Neritimorpha suffered a larger extinction and had a slower recovery during the T–J transition than during the P–T transition.

Our data indicate that the number of gastropod genera reported in the immediate post extinction interval after the Late Triassic crisis (76 gastropod genera in the Hettangian) was larger than in the Early Triassic (40 genera; [90]), after the end-Permian mass extinction. The highest number of first occurrences of gastropod genera after the end-Triassic crisis occurred in the Sinemurian and Pliensbachian time intervals.

Summarized, our data confirm that the end-Triassic mass extinction event caused a profound turnover in gastropods, even in comparison to the greatest Phanerozoic mass extinction event at the end of the Permian. However, the effects of both events were similar in many aspects, and it is currently difficult to conclude which of these events was eventually more important.

## Conclusions

Gastropods lost 56% of genera/subgenera during the end-Triassic mass extinction event, which was much more than the average loss of marine life (46.8%; [18]. Within the phylum Mollusca, gastropods suffered also much more extinction than bivalves, which lost merely 43.4% of genera, but less than ammonoids, which were nearly annihilated. Moreover, we found strong differences among the different subclasses of gastropods. Neritimorphs were most heavily affected and lost 72.7% of the Rhaetian genera, whereas heterobranchs remained nearly unaffected (11% loss) and were blessed with high originations of genera in the Hettangian (43% new genera). The end-Triassic mass extinction therefore paved way for the post-Triassic diversification of this subclass, which is today one of the most species-rich taxon in the Gastropoda.

We infer three traits that correlate with differences in the extinction magnitude among gastropods: (1) Larval development, (2) temperature preference, and (3) flexibility of the mantle attachment. Moreover, we identified two additional traits that may underlay the differences in the extinction magnitude among the mollusc classes classes Cephalopoda (represented by Ammonoidea), Gastropoda, and Bivalvia: (4) shell mineralogy, and (5) locomotion activity. We suggest three potential kill mechanisms that could account for these correlations:

1. A rise in seawater temperature, which favoured species with a physiological preference for high temperatures (Heterobranchia) and harmed species with high energetic demands due to active locomotion (ammonoids versus gastropods, gastropods versus bivalves, and bivalves with high burrowing rates versus stationary or slow moving bivalves).
2. Ocean acidification, which harmed molluscs with purely aragonitic shells (ammonoids and gastropods) more than bivalves that include taxa with a bimineralic shell, and which put infaunal gastropods with the ability to cover their shell by an expanded mantle (Heterobranchia) at a selective advantage over epifaunal gastropods with an inflexible mantle.
3. Extinction of marine plankton, which put heterobranchs with a short pelagic larval phase and the ability to feed on dissolved organic matter and detritus at a selective advantage over the other subclasses.

We note that these interpretations rely on indirect evidence for many of the traits and are therefore preliminary. However, we also note that the inferred kill mechanisms fit very well to the present consensus about the ultimate cause of the end-Triassic mass extinction (CAMP-volcanism) and corresponding environmental changes, in particular CO_2_ induced global warming and ocean acidification.

Finally, we infer from our analysis that the Late Triassic marine mass extinction event caused a profound turnover in early Mesozoic gastropod communities worldwide and catalysed a major radiation of this clade in the Jurassic.

## Supporting information

S1 File List of publications that were evaluated for the analyses. (PDF)

S1 Table Occurrences of gastropod genera and subgenera from the Norian to the Pliensbachian. Occurrences inferred from the range-through assumption are highlighted.
(XLSX)

S2 Table Occurrences of bivalve genera and subgenera from the Norian to the Sinemurian. (XLSX)

S3 Table Palaeobiogeographic distribution of gastropod genera and subgenera during the Norian-Rhaetian and corresponding references. (XLSX)

S4 Table Height (in mm) of *Cylindrobullina* species reported during the studied time interval. (XLSX)
