# Supplementary material for: Gastropods underwent a major taxonomic turnover during the end-Triassic marine mass extinction event
Source: PLoS One. 2022 Nov 2;17(11):e0276329. doi: 10.1371/journal.pone.0276329 (PMC9629647; doi:10.1371/journal.pone.0276329)
Supplement: S1 File — (PDF) [file pone.0276329.s001.pdf]

References

1. Bandel K. Über triassische “Loxonematoidea” und ihre Beziehungen zu rezenten und paläozoischen Schnecken: Paläontologische Zeitschrift. 1991; 65: 239–268.
2. Bandel, K. Platyceratidae from the Triassic St. Cassian Formation and the evolutionary history of the Neritomorpha (Gastropoda). Paläontologische Zeitschrift. 1992; 66(3/4): 231-240.
3. Bandel K. Caenogastropoda during Mesozoic times. Geologica Scripta. 1993a; 2: 7–15.
4. Bandel K. Trochomorpha aus der triassischen St. Cassian Formation (Gastropoda, Dolomiten). Ann. Naturhist. Museum Wien. 1993b; 95: 1-99.
5. Bandel K. Comparison of Upper Triassic and Lower Jurassic Gastropods from the Peruvian Andes (Pucará Group) and the Alps (Cassian Formation). Palaeontographica. 1994; 233: 127–160.
6. Bandel K. How far can gastropod groups be traced in the geological record. In: Unitas Malacologia, Twelfth International Malacological Congress, Vigo 1995. A Guerra, E Rolán, F. Rocha (editors). 1995: 297.
7. Bandel K. Some heterostrophic gastropods from Triassic St. Cassian Formation with a discussion of the classification of the Allogastropoda. Paläontologische Zeitschrift. 1996; 70 (3/4): 325-365.
8. Bandel K. Living fossils among tiny Allogastropoda with high and slender shell from the reef environment of the Gulf of Aqaba with remarks on fossil and recent relatives. Mitt. Geol.-Paläont. Inst. Univ. Hamburg, Heft. 2005; 89: 1-24, 71.
9. Bandel K. Relationships of the Triassic Eucycloidea Koken, 1897 (Mollusca, Gastropoda) to modern genera such as Pagodatrochus, Calliotropis and Euchelus, based on morphology of the early shell. Bulletin of Geosciences. 2010; 85(3): 435–486
10. Bandel K, Gründel J, Maxwell P. Gastropods from the upper Early Jurassic/early Middle Jurassic of Kaiwara Valley, North Canterbury, New Zeland. Freiberger Forschungshefte. 2000; C 490: 67–132.
11. Bayle E, Coquand H. Mémoire sur les Fossiles recueillis dans le Chili par M. Ignace Domeyko et sur les terrains auxquels ils appartiennent. Mémoires de la Societé Géologique de France. 1851; 2/4: 1-47.
12. Begg JG, Grant Mckie JA. New Zealand and New Caledonian Triassic Pleurotomariidae (Gastropoda, Mollusca). Journal of the Royal Society of New Zealand. 2003; 33(1): 223-268.
13. Behrendsen O. Zur Geologie des Ostabhanges der argentinischen Cordillere. Teil I. Zeitschrift der Deutschen Geologischen Gesellschaft. 1891; 43: 369-420.
14. Behrendsen O. Contribución a la geología de la pendiente oriental de la Cordillera Argentina. Actas de la Academia Nacional de Ciencias (Córdoba). 1922; 7: 161–227.

15. Benhamou M., Conti M.A, Elmi S, Monari S. Étude paléontologique et dynamique d’un intervalle condensé à gastropodes dans les calcaires à grands bivalves du Pliensbachien (Ouarsensis, Algérie). GeoActa. 2003 ; 1 : 139–152.
16. Blodgett BR, Frýda J, Stanley GD. Delphinulopsidae, a new neritopsoidean gastropod family from the Upper Triassic (upper Carnian or lower Norian) of the Wallowa terrane, northeastern Oregon. Journal of the Czech Geological Society. 2001; 46/1-2: 221-232.
17. Bouchet P, Rocroi JP, Hausdorf B, Kaim A, Kano Y, Nützel A, Parkhaev P, Schrödl M, Strong EE, et al. Revised classification, nomenclator and typification of gastropod and monoplacophoran families. Malaocologia. 2017; 61(1-2): 1-495.
18. Bourrouilh R. Gastéropodes du Lias inférieur et moyen du domaine atlasique marocain. Notes et Mémoires du Service Géologique du Maroc. 1966: 25-73.
19. Burckhardt C. Profils géologiques transversaux de la Cordillère Argentino−Chilienne. Stratigraphie et tectonique. Anales del Museo de La Plata, Sección Geología y Mineralogía. 1900 ; 2: 1–136.
20. Burckhardt C. Le Lias de la Piedra Pintada (Neuquén). III. Sur les fossilesmarines du Lias de la Piedra Pintada, avec quelques considérations sur l’âge et l’importance du gisement. Revista delMuseo de La Plata. 1902; 10: 243–249.
21. Conrad TA. Remarks on the fossil shells from Chile, collected by Lieutenant Gilliss, with descriptions of the species. In: Gilliss JM, (editor) United States Naval Astronomical Expedition to the Southern Hemisphere during the Years 1849‘50’51’52. Supplementary Papers. A. O. P. Nicholson, Washington DC. 1855 : 282-286.
22. Conti MA, Monari S. Bivalve and gastropod fauna from the Liassic Ammonitico Rosso facies in the Bilecik Area (western Pontides, Turkey), in Farinacci, A., Ager, D.V., and Nicosia, U., eds., Geology and paleontology of western Pontides, Turkey, Jurassic–Early Cretaceous stratigraphy, tectonics and paleogeographical evolution: Geologica Romana. 1991; 27: 245-301
23. Cossmann M. Essais de Paléoconchologie Comparée. Different publishers, Paris. 1895-924; 13 : 3144.
24. Cossmann M. Rectifications de nomenclature: Revue critique de Paléozoologie. 1902; 6: 223.
25. Cossmann, M. Essais de Paléoconchologie Comparée. Septième Livraison: Paris, M. Cossmann, F.R. de Rudeval. 1906: 261.
26. Cossmann M. Essais de Paléoconchologie Comparée: Huitième Livraison: Paris, M. Cossmann, F.R. de Rudeval. 1909 :248.
27. Cossmann M. Contribution a la Paléontologie Franc¸ aise des Terrains Jurassiques. III. Cerithiacea et Loxonematacea. Mémoires de la societé Géologique de France, Peléontologie. 1913; 46: 1–263

28.Cossmann M. Essais de Paleonconchologie comparee, 10. Privately published, Paris. 1916: 292.
29.Cossmann M. Essais de paléoconchologie comparée. Edición del autor, Paris. 1918; 11: 388.
30.Cossmann M. Essais de Paléonconchologie comparée. 1925; 13: 345.
31.Cossmann M. Contribution a la Paléontologie Française des Terrains Jurassiques. Mémoires de la societé Géologique de France, Peléontologie, 1943; 46: 1-263.
32.Cox LR. Moluscos del Triásico superior del Peru (UpperTriassic Mollusca from Peru). Boletın del Instituto Geologico del Peru, Lima. 1949; 12: 150.
33.Cox LR. Jurassic Mollusca from Peru. Journal of Paleontology. 1956; 30(5): 1179-1186.
34.Cox LR, Arkell WJ. A Survey of the Mollusca of the British Great Oolite Series. Palaeontographical Society, London. 1950; Part II: 49–105.
35.Conti MA, Fischer J-C. Preliminary notes on the Aalenian gastropods of Case Canepine (Umbría, Italy). In: « Rosso Ammonitico Symposium » Proceedings: 1981: 137-145.
36.Damborenea SE, Ferrari SM. El género Lithotrochus Conrad (Gastropoda, Vetigastropoda) en el Jura´ sico temprano de Argentina. Ameghiniana. 2008; 45: 197–209.
37.Dareste de la Chavanne J. Fossiles liasiques de la région de Guelma. Bulletin du Service de la Carte Géologique de l'Algérie. 1920; 8: 1–74.
38.D'Orbigny A. Paléontologie Française, Terrain Jurassique II, Gastéropodes, Manson, Paris. 1850–60: 622.
39.Dubar G. Études paléontologiques sur le Lias du Maroc. La fauna Domérienne du Djebel Bou-Dahar, près de Béni-Tajjite, Étude suivie de celle de quelques Mollusques d'autres gisements Marocains, Notes et Mémoires: Maroc, Service Géologique. 1948; 68: 1–250.
40.Edwards CW. Early Mesozoic marine fossils from central Alexander Island. British Antarctic Survey Bulletin. 1980: 49: 33–58.
41.Eudes-Deslongchamps E. Études sur les étages Jurassiques inférieurs de la Normandie: Thèses Présentés a la Faculté des Sciences de Paris. 1864; 296.
42.Ferrari SM. Cosmopolitan Early Jurassic marine gastropods from west−central Patagonia, Argentina. Acta Palaeontologica Polonica. 2009; 54 (3): 449–461.
43.Ferrari, S.M. Early Jurassic Ataphridae (Mollusca: Gastropoda) from Chubut, Argentina: paleogeographic and paleoecologic implications. Ameghiniana. 2011: 48; 63–77.
44.Ferrari SM. The genera Cryptaulax and Procerithium (Procerithiidae, Caenogastropoda) in the Early Jurassic of Patagonia, Argentina. Alcheringa. 2012; 36: 323-336.
45.Ferrari SM. New Early Jurassic gastropods from west central Patagonia, Argentina. Acta Palaeontologica Polonica. 2013; 58: 579-593.
46.Ferrari SM. Patellogastropod and Vetigastopoda (Mollusca, Gastropoda) from the marine Jurassic of Patagonia, Argentina: Historical Biology. 2014; 26: 563–581.

47. Ferrari SM. Early Jurassic marine gastropods from Argentina: palaeobiogeographical analysis based on Vetigastropoda: Journal of Systematic Palaeontology. 2015a; 13: 919–941.
48. Ferrari SM. Systematic revision of Late Triassic marine gastropods from Central Peru: considerations on the LateTriassic/Early Jurassic faunal turnover. Andean Geology. 2015b; 42(1): 71-96.
49. Ferrari SM. Early Jurassic Caenogastropoda and Architectibranchia from the Neuquén Basin, Argentina. Journal of Paleontology. 2017; 91(2): 245-264.
50. Ferrari SM, Bessone S. A new Early Jurassic marine locality from southwestern Chubut Basin, Argentina: Andean Geology. 2015; 42: 349–363.
51. Ferrari SM, Kaim A 2019. Onshore-offshore trend in the evolution of calliotropid gastropods expressed in shell morphology. Journal of Systematic Palaeontology. 2019; 31(1): 115-125.
52. Ferrari SM, Damborenea SE, Manceñido MO, Griffin M. Early Jurassic Trochotomidae (Vetigastropoda, Pleurotomarioidea) from the Neuquén Basin, Argentina. Journal of Paleontology. 2015; 89(2): 331-345.
53. Ferrari M, Blodgett RB, Hodges MS, Hodges CL. Early Jurassic (middle Hettangian) marine gastropods from the Pogibshi Formation (Alaska) and their paleobiogeographical significance. Andean Geology. 2020; 47 (3): 559-576.
54. Ferrari M, Little CTS, Atkinson CTS. Late Toarcian (Lower Jurassic) marine gastropods from the Cleveland Basin, England: systematics, Palaeobiogeography and contribution to biotic recovery from the Early Toarcian Extinction event. Papers in Palaeontology. 2021; 7(2): 885–912.
55. Fischer J-C, Weber C. Revision critique de la Paleontologie Française d'Alcide d'Orbigny, vol II, Gastropodes jurassiques: Paris, Masson. 1997: 300.
56. Fischer JC, Le Nindre YM, Manivit J, Vaslet D. Jurassic gastropod faunas of Central Saudi Arabia. Geo-Arabia. 2001; 6: 63–100.
57. Fischer JC, Rosati F, Raffi S. Sinemurian gastropods from Monte Cucco (Umbria-Marche apennines, Central Italy. Geobios. 2002; 35: 441–456
58. Frýda J, Blodgett RB. Chulitnacula, a new paleobiogeographically distinctive gastropod genus from Upper Triassic strata in accreted terranes of southern Alaska. Journal of the Czech Geological Society. 2001; 46/1-2: 213-220.
59. Frýda J, Blodgett RB, Stanley GD. New neritopsoidean gastropods (Neritimorpha) from the Late Triassic (Late Carnian-Early Norian) of the Wallowa Terrane, northeastern Oregon. Mitteilungen des Geologisch-Paläontologischen, Instituts der Universität Hamburg. 2003; 87: 55-72.

60. Frýda J, Blodgett RB, Stanley GD. New neritopsoidean gastropods (Neritimorpha) from the Late Triassic (Late Carnian-Early Norian) of the Wallowa Terrane, northeastern Oregon. Mitteilungen des Geologisch-Paläontologischen, Instituts der Universität Hamburg. 2003; 87: 55-72.
61. Gatto R, Monari S. Pliensbachian gastropods from Venetian Southern Alps (Italy) and their palaeobiogeographical significance. Palaeontology. 2010; 53: 771–802.
62. Gatto R, Monari S, Neige P, Pinard J-D, Weis R. Gastropods from upper Pliensbachian–Toarcian (Lower Jurassic) sediments of Causses Basin, southern France and their recovery after the early Toarcian anoxic event. Geological Magazine. 2015; 152: 871–901.
63. Gründel J. Procerithiidae (Gastropoda) aus dem Lias und Dogger Deutschlands und Polens: Freiberger Forschungshefte. 1999a; C 481: 1–37.
64. Gründel, J. Truncatelloidea (Littorinimorpha, Gastropoda) aus dem Lias und Dogger Deutschlands und Nordpolens. Berliner Geowissenschaftliche Abhandlungen, Reihe. 1999b; E 30: 89–119.
65. Gründel J. Zygopleuroidea (Gastropoda) aus dem Lias ind Dogger Deutschlands und Nordwestpolens. Paläontologische Zeitschrift. 1999c; 73: 247–259.
66. Gründel J. Archaeogastropoda aus dem Dogger Norddeutschlands und des nordwestlichen Polens. Berliner Geowissenschaftliche Abhandlungen Reihe. 2000; E 34: 205–253.
67. Gründel J. Gastropoden aus dem Jura der südamerikanischen Anden: Freiberger Forschungshefte. 2001a; C492: 43–84.
68. Gründel J. Neritimorpha und weitere Caenogastropoda (Gastropoda) aus dem Dogger Norddeutschlands und des nordwestlichen Polens: Berliner Geowissenschaftliche Abhandlungen, Reihe. 2001b; E 36: 45–99.
69. Gründel J. Die gastropoden der Dogger-Geschiebe Deutschlands und des nordwestlichen Polens. Archiv für Geschiebekunde. 2003a; 4: 129-132.
70. Gründel, J. Gastropoden aus dem Unteren Lias (Ober−Hettangium bis Unter−Sinemurium) Südwestdeutschlands. Stuttgarter Breiträge zur Naturkunde. 2003b; B 340: 1–55.
71. Gründel, J. Die Gattung Discohelix Dunker, 1847 (Gastropoda) und zur Fassung der Discohelicidae Schröder, 1995. Neues Jahrbuch für Geologie und Paläonologie, Monathshefte. 2005: 729–748.
72. Gründel J. Gastropoden aus dem oberen Toarcium/unteren Aalenium (Jura) von Norddeutschland. Paläontologische Zeitschrift . 2007a; 81: 238–253.
73. Gründel J. Gastropoden aus dem unteren Pliensbachium von Feuguerolles (Normandie, Frankreich). Freiberger Forschungshefte. 2007b; C 524/15: 1-34.
74. Gründel, J. Jurassische Gastropoden aus der Betakalkbank (oberes Sinemurium, obere Obtusum-Zone) Südwestdeutschlands. Stuttgarter Beitr. Naturk. 2007c; B370: 1-29.

75. Gründel J. Gastropoden des Pliensbachiums (unterer Jura) aus der Usedom-Senke (Nordostdeutschland). Zitteliana. 2007d; A47: 69-103.
76. Gründel J. Remarks to the classification and phylogeny of the Ataphridae Cossmann, 1915 (Gastropoda, Archaeogastropoda) in the Jurassic. Neues Jahrbuch für Geologie und Paläontologie, Abhandlungen. 2008; 250: 177-197.
77. Gründel J. Neubeschreibung der Gastropodenfauna aus dem Hettangium (unterster Jura) des Kanonenberges bei Halberstadt (Deutschland): Beringeria. 2010; 41: 3-24.
78. Gründel J. Ein ungewöhnlicher Gastropodenfund aus dem Pliensbachium (unterer Jura) Frankens. Geologische Blätter für Nordost-Bayern. 2011; 61: 81-90.
79. Gründel J. Beschreibung einiger Gastropoden aus dem unteren und mittleren Jura des Großherzogtums Luxemburg. Revue de Paléobiologie, Genève. 2012; 31(1): 115-125.
80. Gründel J, Kaim A. Shallow-water gastropods from Late Oxfordian sands in KΠ´by (Pomerania, Poland). Acta Geologica Polonica. 2006; 56 (2): 121-157.
81. Gründel J, Koppka J. Gastropoden aus einem Lias-Geschiebe von Lentschow bei Lassan (Vorpommern, Nordostdeutschland). Archiv für Geschiebekunde. 2007; 4: 643–658.
82. Gründel J, Nützel A. Gastropoden aus dem oberen Pliensbachium (Lias δ2, Zone des Pleuroceras spinatum) von Kalchreuth östlich Erlangen (Mittelfranken): Mitteilungen der Bayerischen Staatssammlung für Paläontologie und Histor. Geologie. 1998; 38: 63–96.
83. Gründel J, Nützel A. On the early evolution (Late Triassic to Late Jurassic) of the Architectibranchia (Gastropoda: Heterobranchia), with a provisional classification: N. Jb. Geol. Paläont. Abh. 2012; 264: 31-59.
84. Gründel J, Nützel A. Evolution and classification of Mesozoic mathildoid gastropods. Acta Palaeontologica Polonica. 2013; 58: 803–826.
85. Gründel J, Nützel A. Gastropoden aus dem oberen Pliensbachium (Amaltheenton-Formation) NE Bayerns (Umgebung von Stauf/Dörlbach/Altdorf) (Franken, Süddeutschland). Zitteliana. 2015; A 55: 45– 76.
86. Gründel J, Kaim A, Nützel A, Little CTS. Early Jurassic gastropods from England. Palaeontology. 2011; 54: 481-510.
87. Haas O. Mesozoic Invertebrate Faunas of Peru: New York, Bulletin of the American Museum of Natural History. 1953: 321.
88. Haszprunar G. The Heterobranchia – a new concept of the phylogeny of the higher Gastropoda: Zeitschrift für zoologische Systematik und Evolutionsforschung. 1984; 23: 15–37.
89. Haszprunar G. On the origin and evolution of major gastropod groups, with special reference to the Streptoneura: Journal of Molluscan Studies. 1988; 54: 367–441.

90.Hayami I, Kase T. A systematic survey of the Paleozoic and Mesozoic Gastropoda and Paleozoic Bivalvia from Japan. University Museum, University Tokyo, Bulletin. 1977; 13: 156

91.Hudleston WH. British Jurassic Gasteropoda. Gasteropoda of the Inferior Oolite. Part I. Palaeontographical Society of London. 1887-1896: 514.

92.Jaworski E. Beiträge zur Kenntnis des Jura in Süd−Amerika. Teil II. Spezieller, paläontologischer Teil. Neues Jahrbuch für Mineralogie, Geologie und Paläontologie Beilage−Band. 1915; 40: 364–456.

93.Jaworski E. Die marine Trias in Südamerika. Part 26. In: Steinmann G (editor) Beiträge zur Geologie und Paläontologie von Südamerika. Neues Jahrbuch fur Mineralogie, Geologie und Paläontologie BB. 1923; 47: 93-200.

94.Jaworski E. Contribución a la paleontología del Jurásico Sudamericano. Publicación de la Dirección General deMinería, Geología e Hidrología, sección Geología. 1925; 4: 1–160.

95.Jaworski E. La fauna del Lias y Dogger de la Cordillera Argentina en la parte meridional de la provincia de Mendoza. Actas de la Academia Nacional de Ciencias (Córdoba). 1926a; 9: 137–316.

96.Jaworski E. Beiträge zur Paläontologie und Stratigraphie des Lias, Doggers, Tithons und der Unterkreide in der Kordilleren im Süden der Provinz Mendoza (Argentinien). Teil I Lias und Dogger. Geologische Rundschau. 1926b; 17a: 373–427.

97.Kaim A. The evolution of conch ontogeny in Mesozoic open sea gastropods: Palaeontologia Polonica. 2004; 62: 3–183.

98.Lycett J. Supplementary Monograph on the Mollusca from the Stonesfield Slates, Great Oolite, Forest Marble and Cornbrash: Monograph Palaeontographical Society. 1863: 129.

99.Mander L, Twitchett RJ, Benton M. Paleoecology of the Late Triassic extinction event in the SW UK. Journal of the Geological Society, London. 2008; 165: 319-332.

100.Marwick J. Divisions and faunas of the Hokonui System (Triassic and Jurassic). New Zealand Geological Survey, Paleontological Bulletin. 1953; 21: 1–141.

101.Mc Roberts C, Blodgett RB. Late Triassic (Norian) Mollusks from the Taylor Mountains Quadrangle, Southern Alaska. Studies by the U.S. Geological Survey in Alaska. U.S Geological Survey Professional Paper. 2002; 1662: 55-75.

102.Monari S, Conti MA, Szabó J. Evolutionary Systematics of the Jurassic Trochoidea: The Familia Colloniidae and subfamily proconulidae. In: J.D. Taylor (editor), Origin and evolutionary radiation of the Mollusca. Oxford University Press (Oxford). 1996: 199-204.

103.Monari S, Marino MC, Conti MA. Palaeobiogeographical significance of some Pliensbachian gastropods from north-eastern Sicily (Italy). Quaderni del Musea Geologico Gemmellaro. 2006; 9: 55-62.

104.Monari S, Valentini M, Conti MA. Earliest Jurassic patellogastropod, vetigastropod, and neritimorph gastropods from Luxembourg with considerations on the Triassic–Jurassic faunal turnover. Acta Palaeontologica Polonica. 2011; 56: 349–384.

105. Monari S, Gatto R, Valentini M. Vetigastropoda and Neritimorpha from the Lower Bajocian of Luxembourg and palaeobiogeography of Aalenian Bajocian (Middle Jurassic) gastropods of western Europe, Journal of Systematic Palaeontology. 2018; 16(6): 449-492.

106. Morris EGS, Lycett J. A monograph of the Mollusca from the Great Oolite, Chiefly from Minchinhampton and the Coast of Yorkshire. Part I, Univalves. Monograph of the Palaeontographical Society of London. 1850: 1–130.

107. Möricke W. Versteinerungen des Lias und Unteroolith von Chile. Neues Jahrbuch für Mineralogie, Geologie und Paläontologie, Beilage Band. 1894; 9: 1–100.

108.Nützel A. The Late Triassic species Cryptaulax? bittneri (Mollusca: Gastropoda: procerithiidae) and remarks on early aspects of the Mesozoic marine revolution. Paläontologische Zeitschrift. 2002; 76(1): 57-63.

109.Nützel A. Recovery of gastropods in the Early Triassic. Comptes Rendus Palevol. 2005; 4: 501 515.

110.Nützel,A, Kießling W. Gastropoden aus dem Amaltheenton (oberes Pliensbachium) von Kalchreuth. Geol. Bl. NO-Bayern. 1997; 47 (1-4): 381-414.

111.Nützel A, Senowbari-Daryan B. Gastropods from the Late Triassic (Norian-Rhaetian) Nayband Formation of Central Iran. Beringeria. 1999; 23: 93-132.

112.Nützel A, Erwin D. New Late Triassic Gastropods from the Wallowa Terrance (Idaho) and their Biogeographic significance. Facies. 2001; 45: 87-92.

113.Nützel A, Erwin D. Late Triassic (Late Norian) gastropods from the Wallowa Terrane (Idaho, USA). Paläontologische Zeitschrift. 2004; 78(2): 361-416.114.

114.Nützel A, Pan H-Z. Late Paleozoic evolution of the Caenogastropoda: larval shell morphology and implicationsfor the Permian/Triassic mass extinction event. Journal of Paleontology. 2005; 79(6): 1175-1188.

115.Nützel A, Gründel J. Two new gastropod genera from the Early Jurassic (Pliensbachian) of Franconia (South Germany). Zitteliana. 2007; A47: 59-67.

116.Nützel A, Gründel J.Early Jurassic (Pliensbachian) gastropods from Franconia, Southern Germany. Palaeontographica, Abt. A: Palaeozoology – Stratigraphy. 2015; 305(1–3): 1–87.

117.Nützel A, Hamedani A, Senowbary-Daryan B. Some Late Triassic Gastropods from the Nayband Formation in Central Iran. Facies. 2003a; 48; 127-134.

118.Nützel A, Blodgett RB, Stanley GD. Late Triassic gastropods from the Martin Bridge Fonnation (Wallowa Terrane) of northeastem Oregon and their paleogeographic significance. - N. Jb. Geol. Paläont. Abh. 2003b; 228: 83 – 100.

119.Nützel A, Mannani M, Senowbary-Daryan B, Yazdi M. Gastropods from the Late Triassic Nayband Formation (Iran), their relationships to other Tethyan faunas and remarks on the Triassic gastropod bady size problem. Neues Jahrbuch für Geologie und Paläontologie - Abhandlungen Band. 2010; 256 (2): 213-228.

120.Nützel A, Aghababalou B, Senowbary-Daryan B. Gastropods from the Norian (Late Triassic) Nayband Formation near Natanz (Iran). Bulletin of Geosciences. 2012; 87(1): 53–65.

121.Nützel A, Kaim A, Grădinaru E. Middle Triassic (Anisian, Bithynian) gastropod from North Dobrogea (Romania) and their significance for gastropod recovery from the end-Permian mass extinction event. Papers in Palaeontology. 2018; 4(4): 477-512.

122.Nützel A, Nose, M. Hautmann, M., Hochleitner, R. (in press). Latest Triassic (Sevatian–Rhaetian) reef carbonates from the Northern Calcareous Alps (Austria), their mollusc dwellers and their fate at the end-Triassic extinction event. Paläontologische Zeitschrift.

123.Pieroni V. The Norian Worthenia-like gastropods (Main Dolomite, Upper Triassic): reclassification of the specimens housed at the “Antonio Stoppani” Natural Museum, Italy. Natural History Sciences. Atti della Società italiana di scienze naturali e del Museo civico di storia naturale di Milano. 2019; 6(1): 37-40.

124.Pieroni V, Monari S, Todd JA. A new caenogastropod from the upper Rhaetian of Lombardy: Palaeobiogeographical history and implications for the Early Jurassic gastropod recovery. Acta Palaeontologica Polonica. 2021; 66(1): 193-206.

125.Riccardi A, Damborenea SE, Manceñido MO, Iglesia Llanos MP. The Triassic/Jurassic boundary in the Andes of Argentina. Rivista Italiana di Paleontologia e Stratigrafia. 2004; 110(1): 69-76.

126.Schulbert S, Gründel J, Nützel A. 2008. Early Jurassic (Upper Pliensbachian) gastropods from the Herforder Liasmulde (Bielefeld, Northwest Germany). Paläontologische Zeitschrift. 2008; 82/1: 17–30.

127.Schulbert C, Nützel A. Gastropods from the Early/Middle Jurassic transition of Franconia (Southern Germany). Bulletin of Geosciences. 2013; 88(4): 723–778.

128.Sha J, Grant-Mackie JA. Late Permian to Miocene bivalve assemblages from Hohxil, Qinghai-Xizang Plateau, China, Journal of the Royal Society of New Zealand. 1996; 26(4): 429-455.

129.Stanley GD. Paleoecology, structure, and distribution of Triassic coral buildups in western North America. University of Kansas Paleontological Contributions. 1979; 65: 68.

130.Stanley G, González-León C, Sandy M, Senowbari-Daryan B, Doyle P, Tamura M, Erwin D, et al. Upper Triassic Invertebrates from the Antimonio Formation, Sonora, Mexico. Journal of Paleontology. 1994; 68(S36): 1-33.

131.Szabó J. Lower and Middle Jurassic Gastropods from the Bakony Mountains (Hungary). Part II. Pleurotomariacea and Fissurellacea (Archaeogastropoda). Annales Historico-Naturales musei Nationalis Hungarici. 1980; 72: 50-71.
132.Szabó J. Lower and Middle Jurassic Gastropods from the Bakony Mountains (Hungary). Part III. Patellacea and Trochacea (Archaeogastropoda). Annales Historico-Naturales musei Nationalis Hungarici. 1981; 73: 55-67.
133.Szabó J. Lower and Middle Jurassic Gastropods from the Bakony Mountains (Hungary). Part IV: Neritacea, Craspedostomacea, Amberleyacea (Archaeogastropoda). Annales Historico−Naturales musei Nationalis Hungarici. 1982; 74: 17–33.
134.Szabó J. Lower and Middle Jurassic Gastropods from the Bakony Mountains (Hungary), Part V. Supplement to Archaeogastropoda; Caenogastropoda. Annales Histroric-Natulares Musei Nationalis Hungarici, Budapest. 1983; 75: 27-46.
135.Szabó J. Two new archaeogastopod genera from the Tethyan Liassic. Annales Histroric-Natulares Musei Nationalis Hungarici, Budapest. 1984; 76: 65-71.
136.Szabó J. Eucyclidae (Eucycloidea), Gastropoda) as a Liassic palaeontological index in the Transdanubian Central Range (Hungary). Hantkeniana. 1995; 1: 67-74.
137.Szabó J. Gastropods of the Early Jurassic Hierlatz Limestone Formation, part 1: a revision of type collections from Austria and Hungarian localities: Fragmenta Palaeontologica Hungarica. 2009; 26: 1–108.
138.Gastropods of the Lower Jurassic Hierlatz Limestone Formation, part 2. Some new archaic type slit-bearing components from the fauna of the Hierlatz Alpe (Hallstatt, Austria) and the Bakony Mts (Hungary). 2016; 33: 3-30.
139.Szabó J. Gastropods of the Lower Jurassic Hierlatz Limestone Formation, part 3. New pleurotomarioideans from the fauna of Hierlatz Alpe (Hallstatt, Austria). Fragmenta Palaeontologica Hungarica. 2017; 34: 9–48.
140.Szabó J. Gastropods of the Lower Jurassic Hierlatz Limestone Formation, part 4. New eucycloidean, trochoidean, neritimorph, and caenogastropod taxa in the fauna of the Hierlatz Alpe (Hallstatt, Austria). Fragmenta Palaeontologica Hungarica. 2018; 35: 61-84.
141.Szabó J. Kericserispira nomen novum, a new substitute name instead of Foveolaria Szabó, 2017 (non Foveolaria Busk, 1884). Fragmenta Palaeontologica Hungarica. 2018; 35: 85.
142.Szabó J, Conti MA, Monari S. Jurassic gastropods from Sicily; new data to the classification of Ataphridae (Trochoidea). Scripta Geologica. 1993; 2: 407-416.
143.Szabó J, Conti MA, Monari S, Wedt J. Gastropods from the Jurassic Neptunian sills of Rocca Busambra (North-Western Sicicly, Italy): Patellogastropoda, Pleurotomarioidea, Scissurelloidea, Fissurelloidea and Eucycloidea. Papers in Palaeontology. 2021; 7: 27-110.

144. Terquem O. Paléontologie de l'étage inférieur de la formation Liasique de la province de Luxembourg, Grand-Duché (Hollande), et de Hettange, du département de la Moselle: Mémoire de la Societé Géologique de France. 1855; 2(3): 219-343.
145. Terquem O, Piette E. Le Lias inférieur de l'est de la France comprenant la Meurthe, la Moselle, le Grand-Duché du Luxembourg, la Belgique et la Meuse: Mémoires de la Société Géologique de France, 2ème Série. 1865; 8: 1-175.
146. Thompson MRA, Turner TH. Early Jurassic fossils from Central Alexander Island and their geological setting. British Antarctic Survey, Bulletin. 1986; 70: 23-39.
147. Tong J, Erwin DH. Triassic gastropods of the southern Quinling Mountains, China. Smithsonian Contribution to Paleobiology. 2001; 92: 1-47.
148. Vitón Í, Comas-Rengifo MJ, Paredes R. Early Jurassic (Sinemurian) gastropods from the Lusitanian Basin (west of Portugal). [Gasterópodos del Jurásico Inferior (Sinemuriense) de la Cuenca Lusitánica (oeste de Portugal)]. Spanish Journal of Palaeontology. 2020; 35 (2): 147-166.
149. Weaver C. Paleontology of the Jurassic and Cretaceous of West Central Argentina. Memoirs of the University of Washington. 1931; 1: 1-469.
150. Wenz W. Gastropoda. Prosobranchia In: O.H. Schindewolf (ed.), Handbuch der Paläozoologie. Verlag von Gebrüder Borntraeger, Berlin. 1938-1944; 6: 1639.
